# Manufacturing and Application of Low-Cost Potting Adhesive with High Thermal Conductivity

**DOI:** 10.3390/ma18215011

**Published:** 2025-11-03

**Authors:** Junxiang Wang, Shiwei Zhang, Caiman Yan, Hongming Li, Shubin Yin, Yong Tang

**Affiliations:** 1 Intelligent Manufacturing Engineering Laboratory of Functional Structure and Device in Guangdong, School of Mechanical and Automotive Engineering, South China University of Technology, Guangzhou 510640, China; 2 Shenzhen Key Laboratory of High Performance Nontraditional Manufacturing, College of Mechatronics and Control Engineering, Shenzhen University, Shenzhen 518000, China

**Keywords:** epoxy resin, line motor, thermal conductivity, thermal management

## Abstract

The air-cored linear synchronous motor (ACLSM), characterized by high precision and stability, is widely applied in high-precision manufacturing. However, due to the absence of an iron core, the windings must be fixed with low thermal conductivity epoxy-based potting adhesive, leading to poor heat dissipation and significant temperature rise, which risks the motor’s lifespan and accuracy. To improve heat dissipation in ACLSM, this research proposes a multi-scale filler-based strategy to enhance the thermal conductivity of the adhesive. A series of comprehensive characterizations and thermal tests demonstrates the effectiveness of this approach. The results demonstrate that the BN-AlN sample exhibits superior thermal conductivity of 1.182 W/m·K at 25 wt% filler loading, a 48.7% enhancement over commercially adhesive 381-4DZ, with only a 38% increase in cost. Meanwhile, it possesses superior electrical insulation properties and appropriate hardness, making it highly suitable for the potting of ACLSM windings. The winding encapsulating with the modified adhesive achieves a maximum temperature reduction of 8.82 °C, while improving temperature uniformity by 29.8%, confirming its exceptional thermal management capability.

## 1. Introduction

High-precision machine tools (HPMTs) are essential for ensuring product performance and manufacturing accuracy [1,2]. The air-cored linear synchronous motor (ACLSM), known for its high precision, stability, and transmission efficiency [3,4], is a key component in delivering the required accuracy for HPMTs [5,6]. However, the absence of an iron core necessitates the use of an epoxy resin to fix the windings. The low thermal conductivity of epoxy resin, combined with poor cooling conditions, leads to insufficient heat dissipation in ACLSM. Consequently, an excessive temperature rise becomes inevitable, impacting motor performance and lifespan severely [7,8]. Therefore, addressing the heat dissipation challenge in ACLSM is critical.

Liquid cooling is a common approach to improving ACLSM heat dissipation, efficiently dissipating heat of windings through the cooling plate [9]. However, the cooling plate only contacts one side of the winding (near side of the winding, NSW), leaving the other side (far side of the winding, FSW). The heat generated by FSW must be transferred to the cooling plate through copper wires and epoxy resin over a long distance, suggesting inferior heat dissipation conditions. In the previous work, we presented a UTVC-based ACLSM structure to enhance the heat dissipation [10]. However, limited by the thermal conductivity of potting adhesive, the heat dissipation of ACLSM cannot be improved by optimizing the heat transfer performance of UTVC infinitely. Therefore, improving the thermal conductivity of potting adhesive is the key to further improving the heat dissipation of ACLSM.

Enhancing the thermal conductivity of epoxy-based adhesives through filling composites is a simple and cost-effective method that is of concern to researchers [11,12]. To achieve high thermal conductivity at low filler content, researchers have explored filler functionalization [13,14], compounding [15,16], ordered orientation [17,18], and the construction of 3D structural frameworks [19,20]. Considering the balance between thermal conductivity and cost, using functionalized and composite fillers is the preferred strategy [21]. For instance, Zeng et al. [22] prepared boron nitride (BN) nanosheet/cellulose nanotube nanocomposites through ultrasonic dispersion and vacuum filtration, achieving high thermal conductivity at 25 wt% BN. Similarly, Yu et al. [23] synthesized hexagonal BN-based epoxy resin nanocomposites via the sol–gel method, which exhibited improved thermal stability and oxidation resistance.

Currently, most research on enhancing the thermal properties of epoxy resin focuses on rotary motors, employing single-component composites typically [24,25,26]. For example, Chen et al. [27] improved the thermal conductivity of potting adhesive using BN, reducing motor winding temperatures by 18.6 °C under liquid cooling conditions. Chang et al. [28] prepared Al_2_O_3_ hybrid resin with high thermal conductivity by the sol–gel method and polymerization method. Compared with traditional motors, the output power of the motor under this scheme increased by 29.6%. However, there is a lack of research on improving epoxy resin thermal conductivity using multi-component composites for motors, particularly for ACLSM. The epoxy resin used in ACLSM must exhibit excellent thermal, mechanical, and insulating properties simultaneously [29]. h-BN, which meets these requirements, is an ideal candidate for improving the thermal conductivity of ACLSM potting adhesive [30,31]. Additionally, incorporating fillers with different dimensional structures creates additional thermal pathways in the vertical direction, further enhancing the thermal conductivity of the potting adhesive. Low-dimensional ceramic fillers, such as Al_2_O_3_ and AlN, are suitable for improving vertical heat transfer [32].

Therefore, this paper proposes a multi-scale filler-based strategy to enhance the thermal conductivity of epoxy potting adhesive. Functionalized h-BN and AlN/Al_2_O_3_ powders were incorporated to construct thermal pathways, improving the overall thermal conductivity of the adhesive. The addition of multi-particle-size 0D fillers not only enables the formation of more thermal conduction paths between 2D fillers but also reduces the generation of internal defects in the material, ensuring the thermal and mechanical properties of the composite material. The effectiveness of the presented strategy was verified through a series of characterizations and experiments, followed by an evaluation of the improved adhesive’s impact on ACLSM heat dissipation. The methods and experimental settings are described in Section 2, while Section 3 provides the results and discussions. The conclusions are summarized in Section 4.

## 2. Methods and Experimental Settings

### 2.1. Materials

The materials include commercially available potting adhesive (381-4DZ, Zhejiang Rongtai Technology Enterprise Co., Ltd., Jiaxing, China), accelerator, curing agent (Zhejiang Rongtai Technology Enterprise Co., Ltd., Jiaxing, China), silane coupling agent (KH-550, Jinan Xingfenglong Chemical Co., Ltd., Jinan, China), anhydrous ethanol (Shanghai Titan Scientific Co., Ltd., Shanghai, China), 1 μm h-BN, 1 μm and 10 μm spherical Al_2_O_3_, 1 μm and 10 μm AlN (Suzhou Yuante New Materials Co., Ltd., Suzhou, China).

### 2.2. Sample Preparation

**Figure 1:**
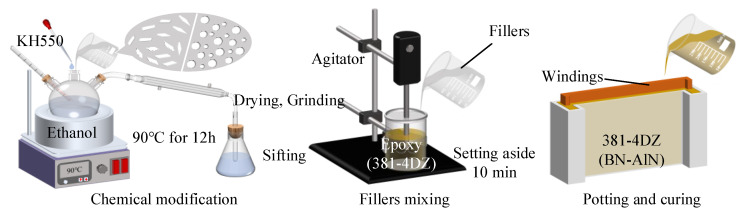
Schematic diagram of the preparation of the BN-AlN-based epoxy sample.

Figure 1 illustrates the process of improving the thermal performance of 381-4DZ through a multi-scale filler-based strategy and implementing ACLSM winding encapsulation. Firstly, the fillers are subjected to surface modification, with h-BN as a representative example: the filler, ethanol, and KH-550 solution were added to a three-necked flask separately for the modification treatment. The mass ratio of ethanol, fillers, and silane coupling agent was 125:25:1. The mixture was heated at 90 °C for 12 h, then cooled to room temperature (~25 °C), and filtered to obtain the powder sample. Finally, the sample was dried at 80 °C in a vacuum drying oven for 12 h, ground, and sieved.

In filler mixing, the ratios of different sizes of components are determined based on the close-packing model:

(1) $$U(D_{p})=100\times \frac{D_{p}^{n}-D_{\min}^{n}}{D_{\max}^{n}-D_{\min}^{n}}$$

where *U*(*D_p_*) is the cumulative particle percentage, *D_p_* is the measured diameter of the fillers, *D*_max_ and *D*_min_ are the maximum and minimum measured diameters of all fillers, respectively. *n* is the grading coefficient, which equals 0.37 in the closest packing state.

**Figure A1:**
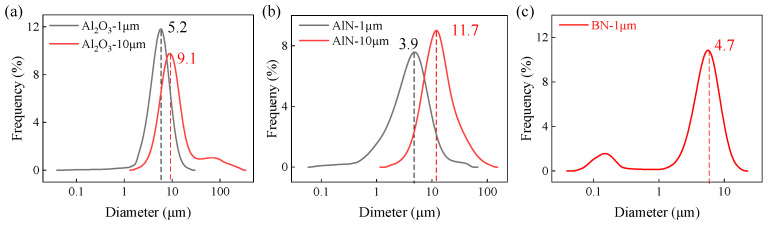
The curve of filler particle size distribution: (**a**) Al_2_O_3_, (**b**) AlN and (**c**) BN.

Based on Equation (1), the optimal mixing ratios are 6:4 for 10 μm AlN and 1 μm AlN, and 7:3 for 10 μm Al_2_O_3_ and 1 μm Al_2_O_3_. (The test results of the filler particle size are presented in Appendix A, Figure A1, and Appendix A, Table A2). The preparation of composites containing h-BN and AlN (BN-AlN) is as follows: Firstly, 1 μm AlN and 10 μm AlN are mixed in the specified proportions and then combined with h-BN powder. Subsequently, the powder mixture is added to 381-4DZ adhesive and mechanically stirred for 10 min by agitation (2000 rpm, LC-ES-200SH, Lichen Instrument Technology Co., Ltd., Changsha, China) to achieve preliminary mixing. Next, a measured amount of accelerator and curing agent is added and mechanically stirred for another 20 min to obtain a homogeneous mixture. The solution is then poured into a mold containing the windings and stands for 10 min to remove bubbles. Finally, the mixture is cured in a vacuum oven at 90 °C for 6 h. Repeating the above procedures, samples with dimensions of 70 × 40 × 10 mm were prepared for characterization and testing. Meanwhile, to evaluate the impact of fillers on the thermal performance of 381-4DZ at different mixing scales, the above steps were repeated separately to prepare samples with only AlN/Al_2_O_3_.

To investigate the effects of filler loadings and filler ratios on epoxy-based potting adhesive, an orthogonal experimental protocol was designed. Based on the h-BN: AlN/Al_2_O_3_ ratio of 4:1, the filler loadings were initially varied from 5 wt% with 5 wt% increments up to 30 wt% to determine the optimal filler loadings. Subsequently, the ratio between h-BN and AlN/Al_2_O_3_ was systematically adjusted across multiple gradients (1:9, 1:4, 1:2, 1:1, 2:1, 4:1, 9:1) to establish the optimal filler based on the optimal filler loadings.

### 2.3. Characterizations

The thermal conductivity of the samples was measured by a Hot Disk thermal conductivity tester TPS2500 (Hot Disk AB, Gothenburg, Sweden). Hardness was measured with a Shore hardness tester (model LX-D). A tungsten filament scanning electron microscope (S-3700N, HITACHI, Tokyo, Japan) was employed to analyze the microstructure and elemental distribution. Fourier Transform Infrared Spectroscopy (Nicolet IS50, Thermo Fisher Scientific, Waltham, MA, USA), and a multi-position automatic sample loading X-ray diffractometer (PANalytical X’pert Powder, Almelo, Netherlands. conventional optical path, wavelength of 0.154 nm, 2θ > 60°, scanning angle range of 5–90°) were used to analyze the structures of modified h-BN, AlN, and Al_2_O_3_ powders. The particle sizes of AlN and Al_2_O_3_ powders were measured by a HORIBA LA960S (HORIBA, Kyoto, Japan) laser particle size analyzer. The volume resistivity of the sample was tested by the RK2670 (Chinarek, Shenzhen, China) fully automatic dielectric strength tester. The thermal stability of the composite material was tested by TG-DSC (STA449F3, NETZSCH, Selb, Germany). The testing conditions were set as follows: nitrogen atmosphere, temperature range from 30 °C to 800 °C, and heating rate of 20 °C/min.

### 2.4. Thermal Test of ACLSM

**Figure 2:**
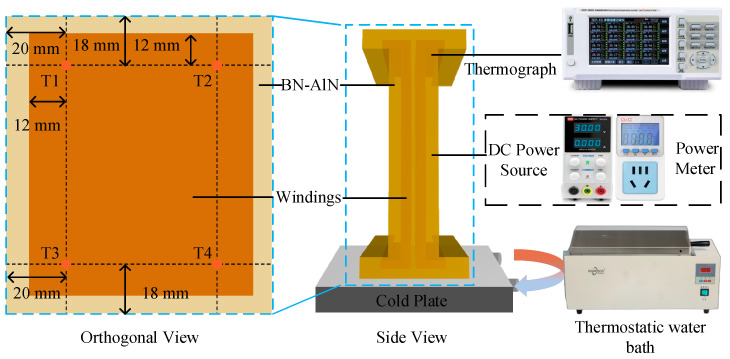
Analog thermal test of ACLSM.

Due to the challenge of monitoring winding temperatures in an operating ACLSM, a simplified simulation test platform was developed to evaluate the thermal performance of the proposed strategy, as shown in Figure 2. In the test, the three-phase windings of the ACLSM were reduced to two single-phase windings to simplify the assembly process and reduce the complexity of adjusting the power device. The procedures involved connecting the windings of the same phase on one side in series, while the windings on the opposite side were connected in parallel. The test system consists of a heating module, a cooling module (including a liquid-cooled plate and a constant-temperature water tank), a data acquisition device (TCP-500X, Hangzhou Huipu Instrument Co., Ltd, Hangzhou, China), and two simulated windings. To monitor the winding temperature in real-time, four type K ultra-fine point thermocouples (Kaipusen Co., Yancheng, China) were installed on the surface of the windings before potting.

During the tests, a heat-conducting silicone grease (5 W/m·K) was evenly applied between the windings and the liquid-cooled plate, while other surfaces were covered with thermal insulation cotton (*k* < 0.2 W/m·K) to minimize the effect of natural convection. The heating power started at 5 W, increasing in 5 W increments after stabilization until the maximum temperature exceeded the protective threshold (110 ± 2 °C). In this experiment, the steady state of the windings was defined as a temperature change of no more than 0.5 °C within one minute. At the equilibrium stage, the temperature distribution of the ACLSM primary was recorded by an infrared thermal imaging camera (FLIR E8-XT, Wilsonville, OR, USA).

Based on the simulation test platform, the effect of BN-AlN potting adhesive on the winding temperature of the ACLSM was investigated by varying the coolant temperature and flow rate. The coolant temperatures included 20 °C, 30 °C, 40 °C, and 50 °C. The flow rates consisted of 1.6 L/min, 2.0 L/min, 2.4 L/min, and 2.8 L/min. In each experiment, only one variable was changed while the other parameters remained constant, as shown in Table 1.

## 3. Results and Discussion

### 3.1. Morphology of Al_2_O_3_, AlN and h-BN

**Figure 3:**
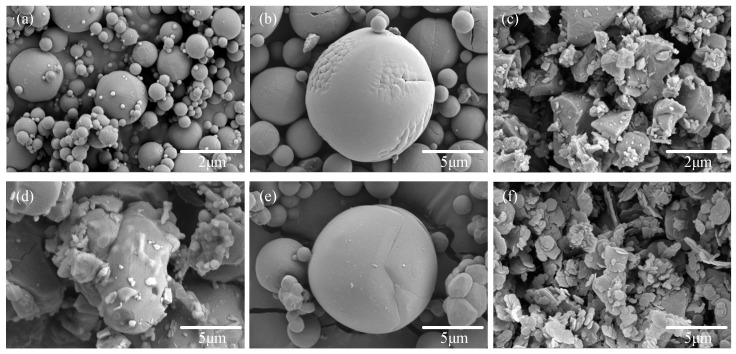
SEM image of Al_2_O_3_, AlN, and BN: (**a**) 1 μm spherical Al_2_O_3_, (**b**) 10 μm spherical Al_2_O_3_, (**c**) 1 μm irregular AlN, (**d**) 10 μm irregular AlN, (**e**) 10 μm spherical AlN, and (**f**) 1 μm BN.

As shown in Figure 3, the SEM images of h-BN, AlN, and Al_2_O_3_ are presented. The surfaces of the 1 μm, 10 μm spherical Al_2_O_3_ and the 10 μm spherical AlN are relatively flat but exhibit minor wrinkles. The 1 μm and 10 μm irregular AlN are distributed in block-like formations with some nanoscale aluminum nitride fragments on the surfaces. The 1 μm h-BN is stacked in plate-like formations, with smoother and flatter surfaces and edges. Due to the high cost of spherical AlN, which is ten times more than irregular AlN, 10 μm irregular AlN was used in subsequent experiments.

### 3.2. Characterization of Al_2_O_3_, AlN, and h-BN

**Figure 4:**
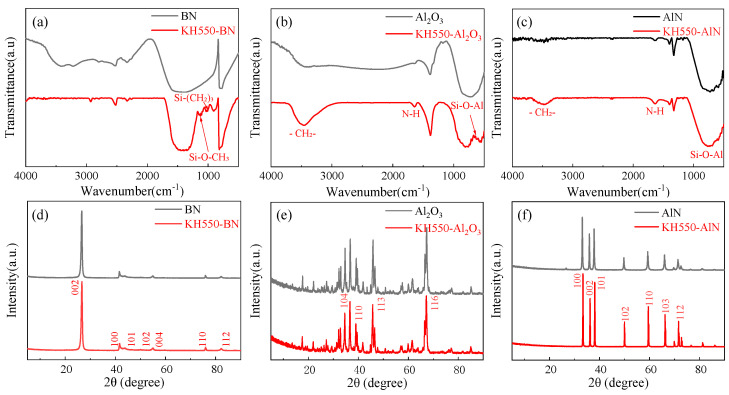
FT-IR and XRD patterns of BN, Al_2_O_3,_ and AlN: (**a**) FT-IR of the modified-BN, (**b**) FT-IR of the modified-Al_2_O_3_, (**c**) FT-IR of the modified-AlN, (**d**) XRD of the modified-BN, (**e**) XRD of the modified-Al_2_O_3_, and (**f**) XRD of the modified-AlN.

The FT-IR and XRD spectra of the three fillers are shown in Figure 4. Figure 4a illustrates the FT-IR spectrum of h-BN, exhibiting characteristic peaks at 783 cm^−1^ and 1373 cm^−1^, corresponding to the in-plane B-N stretching vibration and out-of-plane B-N bending vibration, respectively. The characteristic peaks of Si-(CH_2_)_3_ and Si-O-CH_3_ stretching vibrations are 1021 cm^−1^ and 1104 cm^−1^, respectively, indicating the successful modification of h-BN by KH550 solution. The FT-IR spectrum of treated Al_2_O_3_, as shown in Figure 4b, shows characteristic peaks at 634 cm^−1^, 1651 cm^−1^, and 3462 cm^−1^, corresponding to Si-O-Al, N-H, and -CH_2_-, respectively. The result confirms successful grafting of KH550 onto micrometer-sized Al_2_O_3_. The similar characteristic peaks are observed in treated micron-sized AlN, indicating that AlN was successfully modified by KH550.

The XRD spectra of the three micrometer powders are shown in Figure 4d–f. The diffraction peaks of all three powders remain unchanged after treatment with KH550, indicating that the surface modification by KH550 does not affect the crystal structures of h-BN, Al_2_O_3_, and AlN.

### 3.3. Comprehensive Performance of BN-AlN

**Figure 5:**
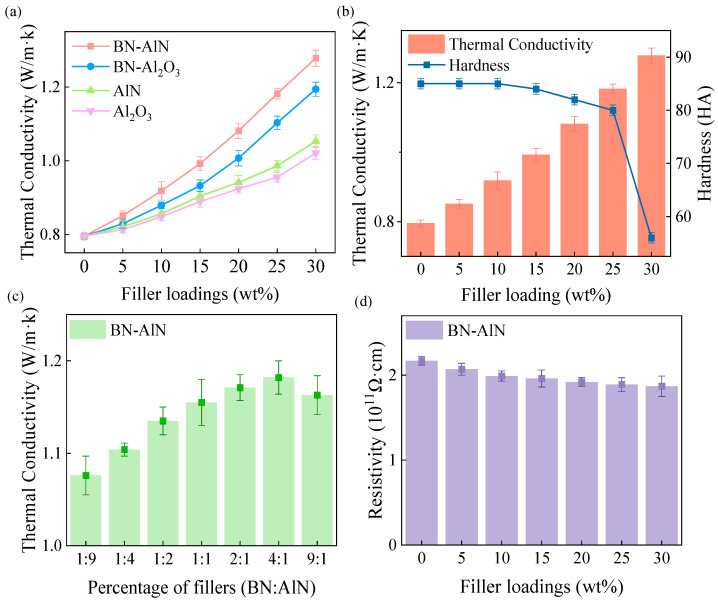
Performance testing of composites: (**a**) Thermal conductivity with different filler loadings, (**b**) Thermal conductivity versus hardness at different filler loadings, (**c**) tThermal conductivity with different filler compounding ratios at 25 wt%, and (**d**) resistivity of composites with different filler loadings.

Figure 5 shows the comprehensive performance of the composites, including thermal conductivity, hardness, and resistivity. The variation in thermal conductivity of the composites at different filler loadings is depicted in Figure 5a. It is evident that the thermal conductivity of the composites increases with the rise in filler content. This is because the incorporation of high thermal conductivity fillers contributes to enhancing the thermal conductivity of the matrix. Furthermore, as the filler loading increases, thermally conductive fillers come into contact within the matrix, forming continuous thermal conduction pathways that enhance heat transfer performance. Notably, at the same filler loading, the BN-AlN composite exhibits the highest thermal conductivity, followed by BN-Al_2_O_3_, while the AlN/Al_2_O_3_ sample shows the lowest value. This improvement stems from the 2D h-BN filler, which constructs interconnected networks among 0D particles, thereby extending heat transfer channels and boosting thermal conduction. Additionally, due to AlN’s inherently higher thermal conductivity compared to Al_2_O_3_, samples incorporating AlN demonstrate superior heat transfer performance. At a 30 wt% filler loading, the thermal conductivity of BN-AlN reaches 1.278 W/m·K, representing a 7.1%, 21.4%, and 25.2% increase compared to the BN-Al_2_O_3_, AlN, and Al_2_O_3_ composites, respectively.

To further clarify the enhancement mechanism of thermal conductivity, the Agari model [33] was used to evaluate the effect of the thermal conduction pathways formed by the filler on thermal conductivity:

(2) $$\log\lambda =V(X_{2}C_{2}\log\lambda_{2}+X_{3}C_{3}\log\lambda_{3}+\cdots X_{n}C_{n}\log\lambda_{n})+(1-V)\log C_{1}\lambda_{1}$$

where *λ* and *λ*_1_ represent the thermal conductivities of the composite material and the polymer matrix, respectively; *λ*_n_ denotes the thermal conductivities of the filler particles; *X_n_* are the statistical fractions of each filler in the total mixed fillers, and the sum of all items equals 1. *C*_1_ is the factor affecting the crystallinity and crystal size of the polymer; *C_n_* are the free factors for the formation of thermal conductive chains by filler particles, reflecting the difficulty level of forming thermal conductive chains by the fillers.

*V* is the volume fraction of the filler in the composite, which is calculated by the following formula:

(3) $$V=\frac{\frac{m_{2}}{\rho_{2}}+\frac{m_{3}}{\rho_{3}}+\cdots +\frac{m_{n}}{\rho_{n}}}{\frac{m_{1}}{\rho_{1}}+\frac{m_{2}}{\rho_{2}}+\frac{m_{3}}{\rho_{3}}+\cdots +\frac{m_{n}}{\rho_{n}}}$$

where m_1_ and *ρ*_1_ represent the mass and density of the matrix material, respectively; m_n_ and *ρ*_n_ denote the mass and density of the filler particles, respectively. In this study, the thermal conductivities and densities of all materials and fillers are provided in Appendix A Table A3.

The calculated *C*_2_ and *C*_3_ values for the BN-AlN scheme, BN-Al_2_O_3_ scheme, AlN scheme, and Al_2_O_3_ scheme are (0.40, 0.44), (0.35, 0.42), (0.22, 0), and (0.35, 0), respectively. The *C*_2_ and *C*_3_ values of the multi-scale filler schemes are all higher than the single-filler schemes, indicating that fillers of different scales can promote each other in forming thermal conductive networks, thereby improving the thermal conductivity of the composites. Compared with the BN-Al_2_O_3_ scheme, the BN-AlN scheme has better *C*_2_ and *C*_3_ values, which means that AlN powder has a more significant promoting effect on the formation of thermal conductive pathways by the main filler (BN powder). Therefore, the BN-AlN composite exhibits higher thermal conductivity.

For industrial motors, hardness is also a critical indicator to evaluate the suitability of potting material. As shown in Figure 5b, the hardness of the composites BN-AlN decreases with the gradual increase in filler loading. This is because higher filler content weakens the interfacial bonding strength between the filler and the epoxy resin, which affects the hardness of composites. At a 30 wt% filler load, the hardness of the composite BN-AlN is only 56 HA, which fails to meet the hardness requirements (80–90 HA) for high-precision ACLSM potting adhesive. Therefore, 25 wt% is an optimal filler loading, providing both a high thermal conductivity (1.182 W/m·K, an increase of 48.7% compared to the matrix 381-4DZ) and satisfied hardness (80 HA).

Based on the determined filler loading, the effect of the h-BN and AlN mixing ratio on the thermal conductivity of the composites was investigated. As shown in Figure 5c, the thermal conductivity of the composites first increased and then decreased as the proportion of h-BN improved. The maximum thermal conductivity is achieved at a h-BN: AlN ratio of 4:1. Because the small size and large specific surface area of h-BN facilitate dispersion within the matrix, enhancing the thermal properties of the composites. However, reducing AlN excessively decreases the thermal conductive pathways in the vertical direction, lowering the thermal conductivity of the composites. Therefore, in this experiment, the h-BN: AlN mixing ratio of 4:1 is the optimal ratio for the filler combination.

The volume resistivity of the composites is presented in Figure 5d. As filler loading increases, the resistivity decreases. Microdefects at the interface between fillers and epoxy resin molecules induce localized electric field concentrations, resulting in defect breakdown under high voltage. In addition, the industrial-grade BN and AlN powders contain certain impurities, which also affect the volume resistivity of the material. However, both BN and AlN possess excellent insulating properties, so they have little impact on the volume resistivity of the composite. At a 25 wt% filler loading, the composite exhibits a volume resistivity of 1.87 × 10^11^ Ω·cm, which is lower than the matrix 381-4DZ but still satisfies the insulation requirement for the DU4-S1-TL120 linear motor (*ρᵥ* ≥ 1 × 10^11^ Ω·cm).

### 3.4. Mapping of BN-AlN

**Figure 6:**
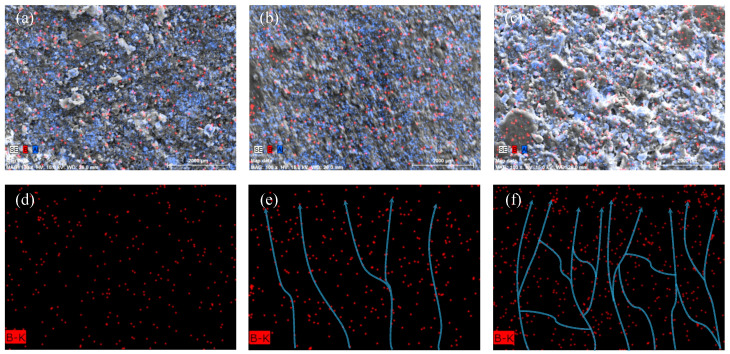
Mapping of BN-AlN: (**a**) mapping of 5 wt% filler loading, (**b**) mapping of 15 wt% filler loading, (**c**) mapping of 25 wt% filler loading, (**d**) B distribution under 5 wt% load, (**e**) B distribution under 15 wt% load, and (**f**) B distribution under 5 wt% load.

Figure 6 shows the distribution of major elements in the sample (The blue and red dots represent the distribution of aluminum and nitrogen elements in the local sample, respectively, while the blue lines represent the possible thermal conduction paths formed under the nitrogen element distribution clusters). Figure 6a–c illustrates the distribution of B and Al elements in the cross-section of the samples at 5 wt%, 15 wt%, and 25 wt% loadings. As the filler load increases, the distribution of B and Al becomes more widespread. The distributions of element B alone are shown in Figure 6d–f. The element content is low and distributed sparsely at a 5 wt% load, making it difficult to form connected thermal conductive pathways. At 15 wt%, h-BN particles gradually connect to form successive thermal conductive pathways. When the load further increases to 25 wt%, more B elements are in contact, forming more successive thermal conductive pathways within the matrix, further enhancing the thermal conductivity of the composite material. Specific elemental contents are shown in Table A1.

### 3.5. Characterization of the Thermal Stability of BN-AlN

**Figure 7:**
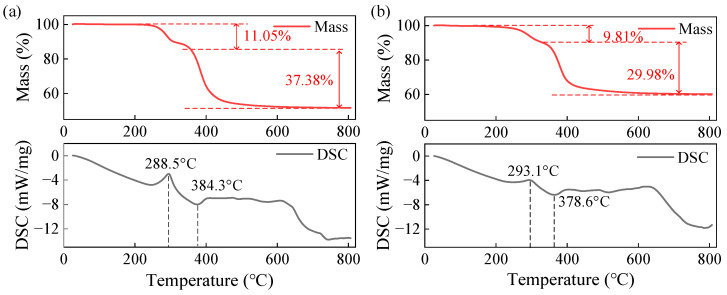
TG-DSC curves of the materials: (**a**) matrix 381-4DZ and (**b**) BN-AlN.

Figure 7 shows the TG-DSC curves of the matrix 381-4DZ and the composite BN-AlN sample. It can be observed that the thermal weight loss curve of the materials exhibits a two-step decomposition process. The first step, occurring between 200 and 400 °C between 200–400 °C, corresponds to the thermal oxidation of the epoxy resin side chains and the carbonization of the main chain. The second step, in the range of 400–600 °C, is the decomposition of the carbonized main chain. At a filler loading of 25 wt%, the initial thermal decomposition temperature of the composite decreases slightly, with the temperature at 1% mass loss dropping from 253 °C to 221 °C. This phenomenon is attributed to several combined factors: the presence of highly active impurities in the ceramic powder, local defect structures between the filler and the matrix, and residual activators from thePlease confirm whether an explanation of the colorful dots and bule lines need to be added to the figure caption. filler modification process. These factors collectively affect the thermal stability of the composite, causing it to start thermal decomposition at a lower temperature. However, the rated operating temperature range of the linear motor is below 110 °C, and the mass loss of the composite at 110 °C is only 0.216% in the thermal weight loss test. Therefore, the BN-AlN composite with 25 wt% filler loading can be considered to have good thermal stability.

### 3.6. Thermal Management Performance Analysis

**Figure 8:**
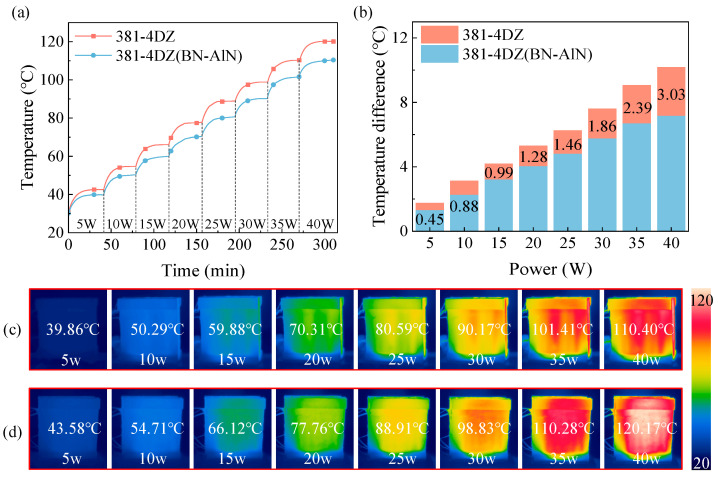
Thermal characteristics of simulated windings with different potting adhesives: (**a**) Maximum temperature of the winding, (**b**) Temperature difference in the winding, (**c**,**d**) are infrared images of ACLSM winding with BN-AlN and 381-4DZ, respectively.

Figure 8 shows the temperature changes in the ACLSM windings with two schemes. As shown in Figure 8a, the BN-AlN scheme consistently exhibits lower temperatures under different power loads. At the rated condition of 35 W, the highest temperature of the BN-AlN scheme is only 101.42 °C, which is 8.82 °C lower than the 381-4DZ scheme. This demonstrates that the composites with high thermal conductivity enhance the heat dissipation of windings. As shown in Figure 8b, the improvement of the BN-AlN scheme in reducing temperature difference becomes more pronounced as the power load increases. At 35 W, the temperature difference in the BN-AlN scheme is 7.15 °C, observing a 29.8% reduction compared to the 381-4DZ scheme.

The infrared images of two schemes under varying power loads are shown in Figure 8c,d. The temperature rises and temperature difference in the windings are significantly decreased with the BN-AlN scheme, demonstrating that the BN-AlN potting adhesive effectively optimizes the heating transfer of the ACLSM windings and enhances thermal management performance.

**Figure 9:**
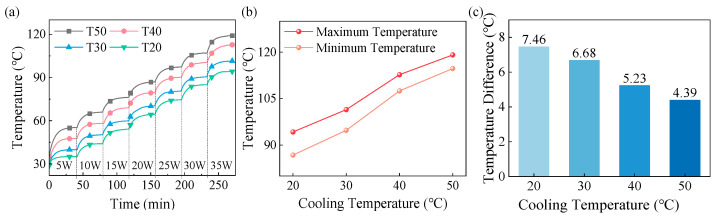
Influences of coolant temperatures on the heating dissipation performance of the BN-AlN scheme: (**a**) Winding temperatures, (**b**) Maximum winding temperatures at rated power, and (**c**) Winding temperature difference at rated power.

Figure 9 illustrates the effect of coolant temperature on the BN-AlN scheme. At the same heating power, increasing the coolant temperature results in higher winding temperature but lower temperature difference. For example, at 35 W, when the coolant temperature is 50 °C, 40 °C, 30 °C, and 20 °C, the maximum temperatures of winding are 119.10 °C, 112.69 °C, 101.44 °C, and 94.22 °C, respectively. The corresponding temperature differences are 4.39 °C, 5.23 °C, 6.68 °C, and 7.46 °C, respectively. Lower coolant temperatures enhance the efficiency of thermal conduction and convection, allowing more heat to be effectively dissipated. However, due to the limited thermal conductivity of the potting compound, the improvement in the efficiency of heat transfer from NSW to the FSW is not significant. Therefore, when the cooling temperature decreases, the heat exchange efficiency of NSW increases, while the heat of FSW is still dissipated through inefficient heat conduction and convection, resulting in an increased temperature difference.

In addition, the winding temperature does not decrease proportionally as the coolant temperature is reduced. At 35 W, when the coolant temperature decreases from 50 °C to 20 °C, the maximum winding temperature decreases by 6.41 °C, 11.25 °C, and 7.22 °C, respectively. Therefore, to balance the cooling performance of the windings and minimize cooling energy consumption, the optimal coolant temperature in this study is set to 30 °C.

**Figure 10:**
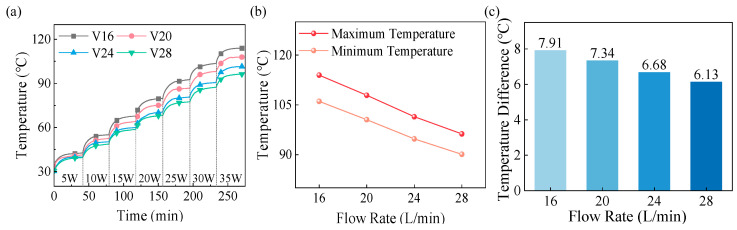
Influences of flow rate on the heating dissipation performance of the BN-AlN scheme: (**a**) Winding temperatures, (**b**) Maximum winding temperatures at rated power, and (**c**) Winding temperature difference at rated power.

Figure 10 shows the effect of the coolant flow rate on the BN-AlN scheme. At the same heating power, increasing the coolant flow rate results in lower winding temperature but higher temperature difference. At the optimal coolant temperature of 30 °C, when the coolant flow rate exceeds 2.4 L/min, the improvement in maximum temperature and temperature difference in winding is less noticeable. When the coolant flow rate is below 2.4 L/min, the maximum winding temperature exceeds the allowable temperature of the winding (110 ± 2 °C). Therefore, at the optimal coolant temperature, the better coolant flow rate is 2.4 L/min.

### 3.7. Economic Analysis

**Figure 11:**
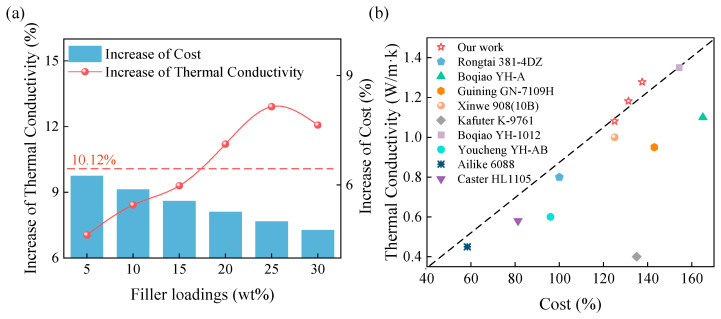
Economic analysis of the BN-AlN: (**a**) The Relationship between the composite’s cost and thermal conductivity increase under different loadings, (**b**) Comparison of economic performance with commercial potting adhesives.

To comprehensively evaluate the commercial viability of BN-AlN, Figure 11 presents an analysis of the economic and thermal performance of BN-AlN, using the cost of 381-4DZ as the 100% standard. In this experiment, the following were defined: the increase in thermal conductivity and the cost of BN-AlN are calculated based on the performance of samples under two adjacent filler loadings, while the average increase in thermal conductivity is obtained by dividing the thermal conductivity of the sample under the maximum loading (30 wt%) by the loading gradient. In this experiment, the average increase in thermal conductivity is 10.12%.

The cost calculation of 381-4DZ is as follows:

(4) $$P_{M}=(1-M)P_{0}+M(0.8P_{BN}+0.2P_{AlN})$$

where *M* refers to the mass fraction of filler loading, *P_M_* represents the cost of BN-AlN material under the filler loading of *M*; *P*_0_, *P_BN_*, and *P_AlN_* correspond to the costs of matrix 381-4DZ, filler BN, and AlN, respectively.

It can be obtained through calculation that when the filler loading of the sample increases by 5 wt% each time, the variation value of each component remains constant, and the cost increase is 5 Yuan per kilogram. As the filler loading amount increases, the cost increases in BN-AlN are 6.25%, 5.88%, 5.60%, 5.26%, 5.00% and 4.76%, respectively. (The costs of main materials are shown in Appendix A Table A4, and the costs of BN-AlN materials under different filler loadings are shown in Appendix A Table A5)

As shown in Figure 11a, it can be observed that at low filler loadings, the increase in thermal conductivity is relatively low, significantly below the theoretical average. As the filler content further increases, the thermal conductivity improvement rises substantially, reaching its peak at 25 wt% loading. Therefore, under a uniform increase in cost, the 25 wt% sample exhibits the highest increase in thermal conductivity and thus the optimal economic efficiency. Combined with the physical performance analysis of the samples in Section 3.3, it can be concluded that the sample with 25 wt% filler loading exhibits the best overall performance and economic characteristics. Figure 11b compares the cost and thermal conductivity of different commercial motor potting adhesives. BN-AlN achieves superior thermal performance at a lower cost. This solution provides a novel strategy for the design of commercial motor potting adhesives.

## 4. Conclusions

In this study, a multi-scale filler-based strategy of epoxy-based potting adhesive for ACLSM thermal management was proposed. By mixing micrometer-sized h-BN and AlN powders into the matrix, a thermal conductive pathway was created to enhance the thermal conductivity of the adhesive. The thermal conductivity of the adhesive was further optimized by adjusting the filler loading and the ratio of h-BN to AlN powders. At a 25 wt% loading and a BN:AlN ratio of 4:1, the thermal conductivity of the BN-AlN reached 1.182 W/m·K, representing a 48.7% increase compared matrix (381-4DZ). Moreover, the presented adhesive exhibits excellent hardness and insulation properties, meeting the assembly requirements of ACLSM. At the optimal ratio, the hardness reaches 80 HA, and the volume resistivity is 1.87 × 1011 Ω·cm. In the simulated temperature test for ACLSM windings under rated power, the highest temperature with the BN-AlN composite decreases by 8.82 °C, and the temperature difference reduces by 29.8%. These excellent performance characteristics indicate that the presented strategy offers a promising solution for heat dissipation in ACLSM, satisfying both thermal management and insulation requirements.

## Figures and Tables

**Table 1:** Orthogonal experiment of flow rate and coolant temperature.

Test	Flow Rate (L/min)	Coolant Temperature (°C)
1	2.4	20
2	2.4	30
3	2.4	40
4	2.4	50
5	1.6	Optimal coolant temperature
6	2.0	Optimal coolant temperature
7	2.8	Optimal coolant temperature

## Data Availability

The original contributions presented in this study are included in the article. Further inquiries can be directed to the corresponding author.

## Abbreviations

The following abbreviations are used in this manuscript:

ACLSM	Air-cored linear synchronous motor
HPMTs	High-precision machine tools
NSW	The one side of winding near the cold plate
FSW	The one side of winding far away the cold plate
BN	Boron nitride
Al_2_O_3_	Aluminum oxide
AlN	Aluminum nitride

## Appendix A

**Table A1:** Elemental contents of B, N and Al.

Filler Loading (wt%)	Content of B (%)	Content of N (%)	Content of Al (%)
0	7.38	3.02	4.77
5	11.19	3.95	5.23
10	16.53	5.46	5.46
15	20.28	6.47	6.01
20	24.53	7.49	6.57
25	27.45	9.69	6.94

**Table A2:** The parameters of filler particle size distribution.

Material	D_50_ (μm)	D_min_ (μm)	D_max_ (μm)
1 μm Al_2_O_3_	5.199	0.078	22.797
10 μm Al_2_O_3_	9.092	1.729	229.075
1 μm AlN	3.909	0.076	44.938
10 μm AlN	11.739	1.729	116.210
1 μm BN	4.722	0.058	17.377

**Table A3:** Density and thermal conductivity of the matrix and filler.

Material	Density (g/cm^3^)	Thermal Conductivity (W/m·K)
Matrix 381-4DZ	1.708	0.795
Al_2_O_3_	3.92	60
AlN	3.26	320
BN	2.29	40

**Table A4:** The costs of main materials.

Material	Cost (Yuan/kg)
Matrix 381-4DZ	80
Al_2_O_3_	50
AlN	200
BN	200

**Table A5:** The costs and the cost increase in BN-AlN under different filler loadings.

Filler Loading (wt%)	Cost (Yuan/kg)	Cost Increase (%)
0	80	6.25
5	85	5.88
10	90	5.60
15	95	5.26
20	100	5.00
25	105	4.76
